# miR-155 inhibits chondrocyte pyroptosis in knee osteoarthritis by targeting SMAD2 and inhibiting the NLRP3/Caspase-1 pathway

**DOI:** 10.1186/s13018-021-02886-5

**Published:** 2022-01-28

**Authors:** Gen Li, Lijun Xiu, Xiaoyun Li, Lisha Ma, Jihui Zhou

**Affiliations:** 1 Department of Spine Surgery, QingDao HuangDao District Central Hospital, Qingdao, 266500 Shandong China; 2 Department of Hand and Foot Surgery, Qingdao Eighth People’s Hospital, Qingdao, 266100 Shandong China; 3 Department of Ophthalmology, Qingdao Eighth People’s Hospital, Qingdao, 266100 Shandong China; 4 Department of Traumatic Orthopedics, Maoming People’s Hospital, No. 101 Weimin Road, Maoming, 525000 Guangdong Province China

**Keywords:** Knee osteoarthritis, miR-155, SMAD2, Pyroptosis, NLRP3, Caspase-1, Lipopolysaccharide, Inflammatory factors

## Abstract

**Objective:**

Knee osteoarthritis (KOA) is based on degenerative pathological changes. miR-155 is involved in regulating KOA. This study estimated the mechanism of miR-155 in mouse KOA chondrocytes.

**Methods:**

Mouse KOA chondrocyte model was established by lipopolysaccharide (LPS) induction and identified through Collagen II immunofluorescence staining and toluidine blue staining. LPS-induced KOA chondrocytes were transfected with miR-155 inhibitor or/and si-SMAD2, followed by the evaluation of miR-155 expression, pyroptosis, the SMAD2/NLRP3/Caspase-1 axis-related protein levels, IL-1β and 1L-18 levels, and cell viability by RT-qPCR, FAM-FLICA Caspase-1 Detection Kit, Western blot, ELISA, and MTT assays, respectively. The binding sites between miR-155 and SMAD2 were predicted online and the binding relationship was verified by dual-luciferase assay.

**Results:**

miR-155 was highly-expressed in LPS-induced KOA chondrocytes. miR-155 knockdown increased cell viability and decreased pyroptotic chondrocytes, and Caspase-1, 1L-1β and 1L-18 levels. miR-155 targeted SMAD2. SMAD2 knockdown partially annulled the effects of miR-155 silencing on inhibiting KOA chondrocyte pyroptosis. NLRP3 pathway was activated in LPS-induced KOA chondrocytes, inhibited after miR-155 knockdown, and activated again after further SMAD2 knockdown. NLRP3 inhibition suppressed Caspase-1, IL-1β, and IL-18 levels and chondrocyte pyroptosis and increased cell viability.

**Conclusion:**

miR-155 knockdown inhibited the NLRP3/Caspase-1 pathway by targeting SMAD2, thus inhibiting mouse KOA chondrocyte pyroptosis.

## Introduction

Osteoarthritis (OA) is a prevalent form of the degenerative joint disorder characterized by synovial inflammation and the gradual degradation of the articular cartilage, and the knee is the major peripheral joint affected causing progressive loss of the function, stiffness and pain [1, 2]. Various personal level of risk factors for OA are identified, including genetic predispositions, sociodemographic characteristics such as female sex, diet-related factors, high bone density/mass, and obesity [3]. Knee OA (KOA) is one of the most prevalent degenerative disorders, which results in disability in the elderly patients [4]. A relevant study demonstrated indirect evidence of the role of chronic inflammation in patients with rheumatoid arthritis in metabolic syndrome and atherosclerosis development [5]. A variety of pro-inflammatory cytokines including interleukin 1 beta (IL-1β) and IL-18 secreted by an increased number of inflammatory cells exacerbate OA [6, 7]. KOA affects 3 compartments of knee joints (patellofemoral, medial, and lateral joint), which usually slowly develops over 10–15 years and interferes with the daily life activities [8]. Therefore, it is necessary to study the pathogenesis of KOA.

Pyroptosis is a kind of pro-inflammatory programmed cell death triggered by inflammasomes, which induces cell rupture and cell content release [9]. Pyroptosis is induced by the typical caspase-1 inflammasomes or caspase-4, caspase-5 and caspase-11 activation caused by cytosolic lipopolysaccharide [10]. The inflammasome biochemical function is to activate caspase-1 that leads to IL-1β and IL-18 maturation and the induction of pyroptosis [11]. The participation of pyroptosis has also reported in OA [12]. But the mechanism is elusive.

In addition, it is reported that tendon, as the medium connecting muscle and bone, can transfer the force generated by muscle to bone, so as to make the joint move or stabilize. Factors such as persistent knee varus or valgus lead to tendon injury, resulting in OA. Various genes and growth factors involved in tendon lesions and tendon healing are reported to be regulated by siRNAs and microRNAs (miRNAs), thus participating in the occurrence and development of OA [13, 14]. Studying the role of miRNAs in joint physiology and pathology may provide reference for the diagnosis, prevention, and treatment of OA [15]. miRNAs are small, single-stranded RNAs, which are related to a variety of diseases, such as cancer, OA and diabetes [6, 16]. miR-155 is reported to be upregulated in lipopolysaccharide (LPS)-induced inflammatory articular chondrocyte [17]. miR-155 participates in pyroptosis by regulating the corresponding proteins [18]. SMAD family member 2 (SMAD2) plays an important role in transporting extracellular signals from the TGF-β superfamily ligands into cell nucleus that cause modulation of a variety of cellular processes, such as cell differentiation, proliferation and apoptosis [19]. CircCDK14 protects against OA by promoting SMAD2 expression [20]. The nucleotide-binding domain-like receptor protein3 (NLRP3)/caspase-1 pathway is involved in the pyroptosis, release and maturation of the inflammatory cytokines mediated by NLRP3 [21]. The activation of the SMAD2/3 pathway can inhibit the transcription of NLRP3, thus reducing the pyroptosis of hypoxic cardiomyocytes [22]. NLRP3 plays a role in synoviocytes from OA joint [23]. However, there is no report at present on whether miR-155 can affect the activation of the NLRP3/Caspase-1 pathway by regulating SMAD2, thus affecting KOA chondrocyte pyroptosis. This study estimated the mechanism of miR-155 on KOA chondrocyte pyroptosis to provide a possible treatment for KOA from the perspective of gene.

## Materials and methods

### Culture and transfection of mouse KOA chondrocytes

The normal mouse KOA chondrocytes (GK1289, Huayueyang Biotechnology, Beijing, China) were cultured in the Dulbecco’s modified eagle medium (DMEM)/F12 medium (Procell, Wuhan, Hubei, China) supplemented with L-glutamine (2.0 mM), penicillin/streptomycin (100 U/mL, 100 mg/mL) (Sigma-Aldrich, St. Louis, MO, USA) and 10% fetal bovine serum (FBS) at 37 °C containing 5% CO_2_.

### Identification of mouse KOA chondrocytes

Collagen II immunofluorescence staining was performed as follows: the monolayer cells were dripped on the cover glass, fixed with 4% paraformaldehyde for 20 min and incubated with 3% H_2_O_2_ (Sinopharm, Beijing, China) at room temperature for 10 min. After that, the cells were rinsed with phosphate buffer saline (PBS) (Hyclone, Logan, UT, USA) for 5 min 3 times and incubated with 5% bovine serum albumin (BSA) (Sigma-Aldrich) for 10 min, and the serum was removed. The cells were added with rabbit Collagen II antibody (ab34712, 1:200, Abcam, Cambridge, MA, USA) at 4 °C overnight. Subsequently, the cells were rinsed thrice with PBS, incubated with biotin-labeled goat anti-rabbit IgG secondary antibody (1:100, A0277, Beyotime, Shanghai, China) for 10 min and rinsed with PBS 3 times. Finally, the cells were cultured with 2,4-diaminobutyric acid, re-stained with hematoxylin (Sinopharm), dehydrated with absolute ethanol and observed under an optical microscope.

Toluidine blue staining was performed as follows: the chondrocytes were seeded in 6-well plates with pre-fixed sterile cover glass. After adhesion, the cells were collected and fixed with 40 g/L polytoluene for 20 min and 75% ethanol for 15 min, and stained with 1% toluidine blue for 2–3 h. Then, the cells were rapidly rinsed with absolute ethanol, cleared with xylene (Sinopharm) and observed under an optical microscope.

### LPS-induced chondrocyte inflammation model

The chondrocyte suspension was transferred to a clean culture flask. The chondrocytes were cultured in an incubator containing 5% CO_2_ at 37 °C and the culture medium was refreshed every 48 h. When the adherent cells covered 90% of the bottle bottom, the chondrocytes were treated with the DMEM containing 10 μg/mL LPS (Sigma-Aldrich) for 6 h [24] and the chondrocyte inflammation model was established.

### Cell transfection and grouping

The mouse knee chondrocytes in the logarithmic growth stage were transfected using Lipofectamine 2000 transfection kit (Invitrogen, Carlsbad, CA, USA). The transfected cells were made into cell suspension using DMEM (10% FBS), seeded in 24-well plates at 1 × 10^5^ cells/well and cultured in a constant temperature incubator at 37 °C with 5% CO_2_ with 95% humidity. The miR-155 inhibitor and its negative control inhibitor NC, and siRNA-SMAD2 and siRNA-NC used for transfection were all provided by Biomics Biotech (Nantong, Jiangsu, China).

The cells were allocated to the following 8 groups: normal group (mouse KOA chondrocytes without any treatment), LPS group (chondrocytes with 10 μg/mL LPS treatment for 6 h), LPS + inhi NC group (cells transfected with inhibitor NC for 48 h and induced with 10 μg/mL LPS for 6 h), LPS + miR inhi group (cells transfected with miR-155 inhibitor for 48 h and induced with 10 μg/mL LPS for 6 h), LPS + miR inhi + si-NC group (cells co-transfected with miR-155 inhibitor and si RNA for 48 h and induced with 10 μg/mL LPS for 6 h), LPS + miR inhi + si-SMAD2 group (cells co-transfected with miR-155 inhibitor and siRNA-SMAD2 for 48 h and induced with 10 μg/mL LPS for 6 h), LPS + miR-inhi + si-SMAD2 + MCC950 group [cells co-transfected with miR-155 inhibitor and siRNA-SMAD2 for 48 h and treated with LPS (10 μg/mL) and NLRP3 inhibitor MCC950 (10 μM) for 6 h [25]], and LPS + miR-inhi + si-SMAD2 + PBS group (cells co-transfected with miR-155 inhibitor and siRNA-SMAD2 for 48 h and treated with LPS (10 μg/mL) and PBS (10 μM) for 6 h).

### Reverse transcription quantitative polymerase chain reaction (RT-qPCR)

The mouse KOA chondrocytes were collected and the total RNA of the cells was extracted using the Trizol reagent. The RNA was reverse transcribed into cDNA using the reverse transcription kit (Takara, Tokyo, Japan). The qPCR was performed using the SYBR® Premix Ex Taq™ II fluorescence quantitative kit for protein level analysis. The miRNAs were synthesized by Sangon Biotech (Shanghai, China). The qPCR of miRNAs was performed. The RNA was reverse-transcribed using the first strand cDNA (Sangon) synthesized by miRNA, and then quantified by miRNA qPCR kit (SYBR Green Method) (Sangon). Table 1 exhibits the primer sequence.

**Table 1:** Primer sequence

Name of primer	Sequences
*miR-155*-F	ATTGCCAATTTCTCTACCAC
*miR-155*-R	AGTAACAGGCATCATACACT
*U6*-F	CTCGCTTCGGCAGCACA
*U6*-R	AACGCTTCACGAATTTGCGT
*SMAD2*-F	CACGCACTTCCGCACATTC
*SMAD2*-R	TAAGGGCGAAAAAGCAGTT
*GAPDH*-F	CAAGGTCATCCATGACAACTTTG
*GAPDH*-R	GTCCACCACCCTGTTGCTGTAG

### Western blot (WB)

The mouse knee chondrocytes were added with protease inhibitor and phosphatase inhibitor. The total protein was isolated using M-PER (Pierce, Rockford, IL, USA) on ice and the protein concentration of the cells was determined using the bicinchoninic acid kit (Thermo Fisher Scientific, Waltham, MA, USA). The protein was isolated using the sodium dodecyl sulfate–polyacrylamide gel electrophoresis at 60 V (30 min) and 110 V (1 h). After electrophoresis, the protein was transferred to the polyvinylidene fluoride (PVDF) membranes using the wet electric transfer method for 2 h in a 4 °C-cold chamber. Then, the PVDF membranes were sealed with 5% skim milk-Tris buffered saline-Tween 20 (TBST) and incubated at room temperature for 1–2 h. The membranes were incubated with primary rabbit monoclonal antibody SMAD2 (ab280888, 1:1000, Abcam), rabbit polyclonal antibody NLRP3 (ab214185, 1:1000, Abcam) and rabbit polyclonal antibody Caspase-1 (ab138483, 1:1000, Abcam) at 4 °C overnight. Then, the membranes were washed thrice with TBST, added with horseradish peroxidase-labeled goat anti-rabbit IgG secondary antibody (1:5000, SSA004, Sino Biological Inc., Beijing, China), incubated at room temperature for 1 h and washed with TBST 3 times. With GAPDH as the internal reference, the membrane was visualized and the data were analyzed.

### Enzyme-linked immunosorbent assay (ELISA)

After 48 h of the transfection, the release levels of inflammatory factors 1L-1β (MLB00C, R&D SYSTEMS, Minneapolis, MN, USA) and 1L-18 (FK-KJ2900, FANKE) in LPS-induced mouse knee chondrocytes were determined using the corresponding Quantikine ELISA kit.

### 3-(4,5-Dimethylthiazol-2-yl)-2,5-diphenyltetrazolium bromide (MTT)

The chondrocytes were seeded in 96-well plates and the plates without chondrocytes were added with DMEM medium as the control. The samples were cultured for 72 h, added with 50 μL 5 mg/mL MTT solution and incubated in an incubator containing 5% CO_2_ at 37 °C for 4 h and the medium was removed. After the samples were dissolved with dimethyl sulfoxide, the optical density (OD) value was measured at 490 nm using a microplate (Thermo Fisher Scientific).

### Pyroptosis detection

The change of chondrocyte pyroptosis was assessed by the double staining of propidium iodide and FAM-YVAD-FMK using the FAM-FLICA Caspase-1 detection kit (ImmunoChemistry, Bloomington, MN, USA). The chondrocytes were stained with green FLICA caspase-1 inhibitor reagent at 37 °C for 60 min and re-stained with Hoechest 33258.

### Dual-luciferase assay

The binding sites between miR-155 and SMAD2 were predicted using the online database (http://starbase.sysu.edu.cn/agoClipRNA.php?source=mRNA). The wild type (WT) or mutant (MUT) of SMAD2 3ʹ-UTR luciferase reporter gene was constructed and cloned into the pMIR vector (RiboBio, Guangzhou, Guangdong, China). The miR-155mimics or mimics NC and the constructed WT or MUT luciferase reporter vectors were co-transfected into normal mouse knee chondrocytes. After 48 h, the fluorescence activity was detected using the luciferase kit (YPH-Bio, Beijing, China).

### Statistical analysis

SPSS21.0 (IBM Corp. Armonk, NY, USA) and GraphPad Prism 8.01 (GraphPad Software Inc San Diego, CA, USA) were used for statistical analysis and mapping of all the experimental data. The data were expressed as mean ± standard deviation. Independent *t* test was used for data comparisons between 2 groups and one-way analysis of variance (ANOVA) was used for data comparisons among multi-groups. Tukey’s multiple comparisons test was used for the post hoc test.* P* < 0.05 was indicative of statistical significance.

## Results

### miR-155 was highly-expressed in LPS-induced mouse KOA chondrocytes

**Fig. 1:**
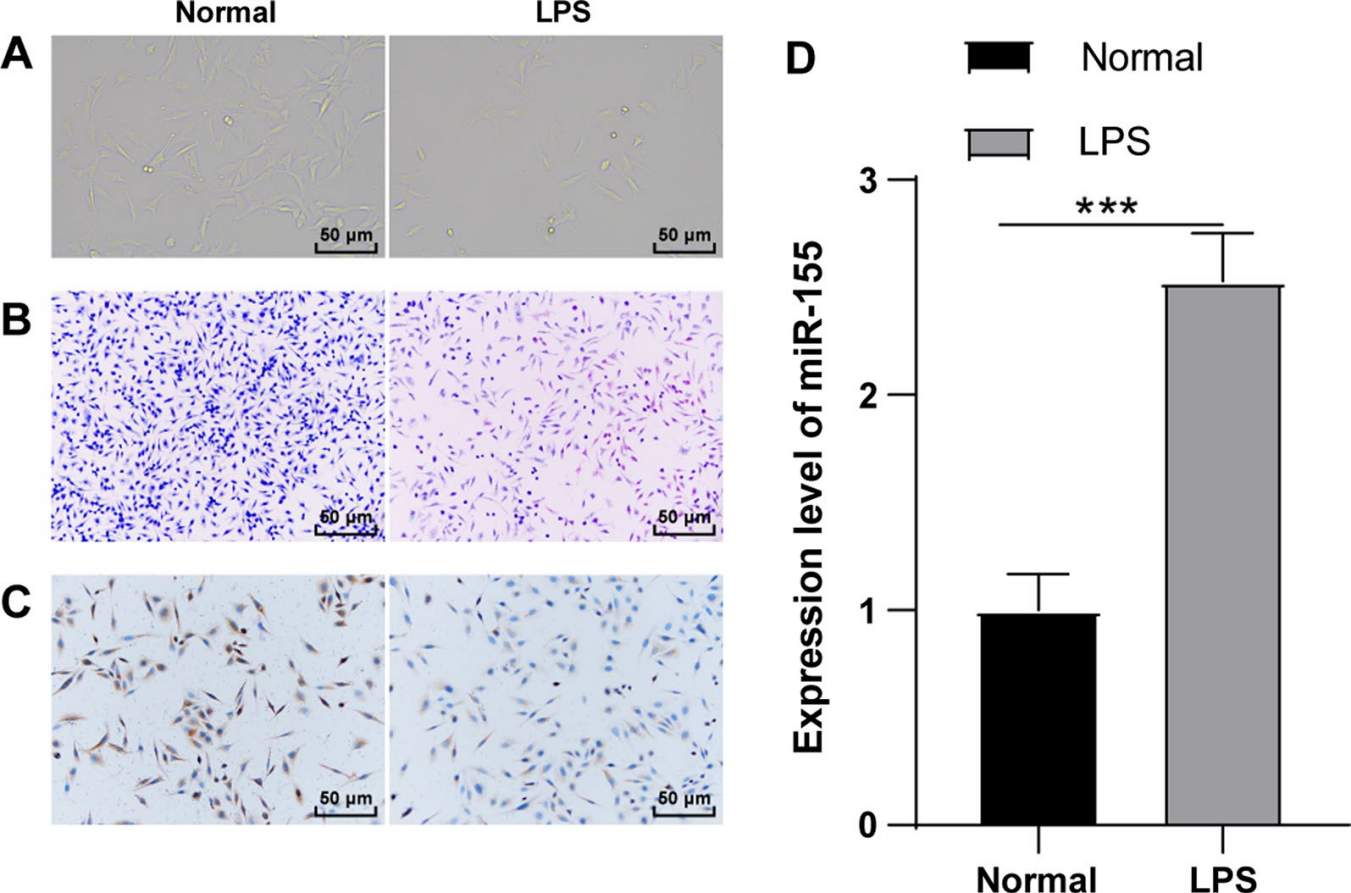
miR-155 was highly expressed in LPS-induced mouse KOA chondrocytes. The mouse knee chondrocyte inflammatory model was established by LPS induction. **A** The cell morphology was observed using an inverted microscope; **B** The cell morphology was observed using toluidine blue staining; **C** The expression of the knee chondrocyte marker Collagen II was detected by immunocytochemistry; **D** The expression of miR-155 was detected using RT-qPCR. The cell experiment was repeated 3 times. The data were expressed as mean ± standard deviation. Independent *t* test was used for data comparisons between 2 groups. *** *P* < 0.001

miR-155 in knee chondrocytes is closely associated with KOA [17]. To explore the mechanism, the The mouse knee chondrocytes were induced by LPS and the chondrocyte inflammation model was established (LPS group). The cell morphology was observed using inverted microscopy and toluidine blue staining. The expression of collagen II (a marker of knee chondrocytes) was detected by immunocytochemistry for mouse knee chondrocyte identification. The normal chondrocytes (normal group) showed adherent growth in polygonal shape in typical paving stone-like shape with clear nucleus and rich cytoplasm; after toluidine blue staining, the cytoplasm was light blue and the nucleus was dark blue; after Collagen II immunochemical staining, the chondrocytes were positive, brown, and the nucleus was yellowish brown. Compared with the normal chondrocytes, after LPS induction, the volume of chondrocytes was increased, the number of vacuoles in the cytoplasm was increased, the volume of nucleus was increased, the morphology of some cells was irregular and the number was relatively reduced, and toluidine blue staining and collagen II immunohistochemical staining were decreased (Fig. 1A–C). miR-155 in knee chondrocytes is closely associated with KOA [17]. The expression of miR-155 in chondrocytes was detected using RT-qPCR. Compared with the normal group, miR-155 in the LPS group was significantly upregulated (*P* < 0.001) (Fig. 1D). Briefly, miR-155 was highly-expressed in LPS-induced KOA chondrocytes and might be involved in KOA occurrence.

### Knockdown of miR-155 inhibited LPS-induced KOA chondrocyte pyroptosis

**Fig. 2:**
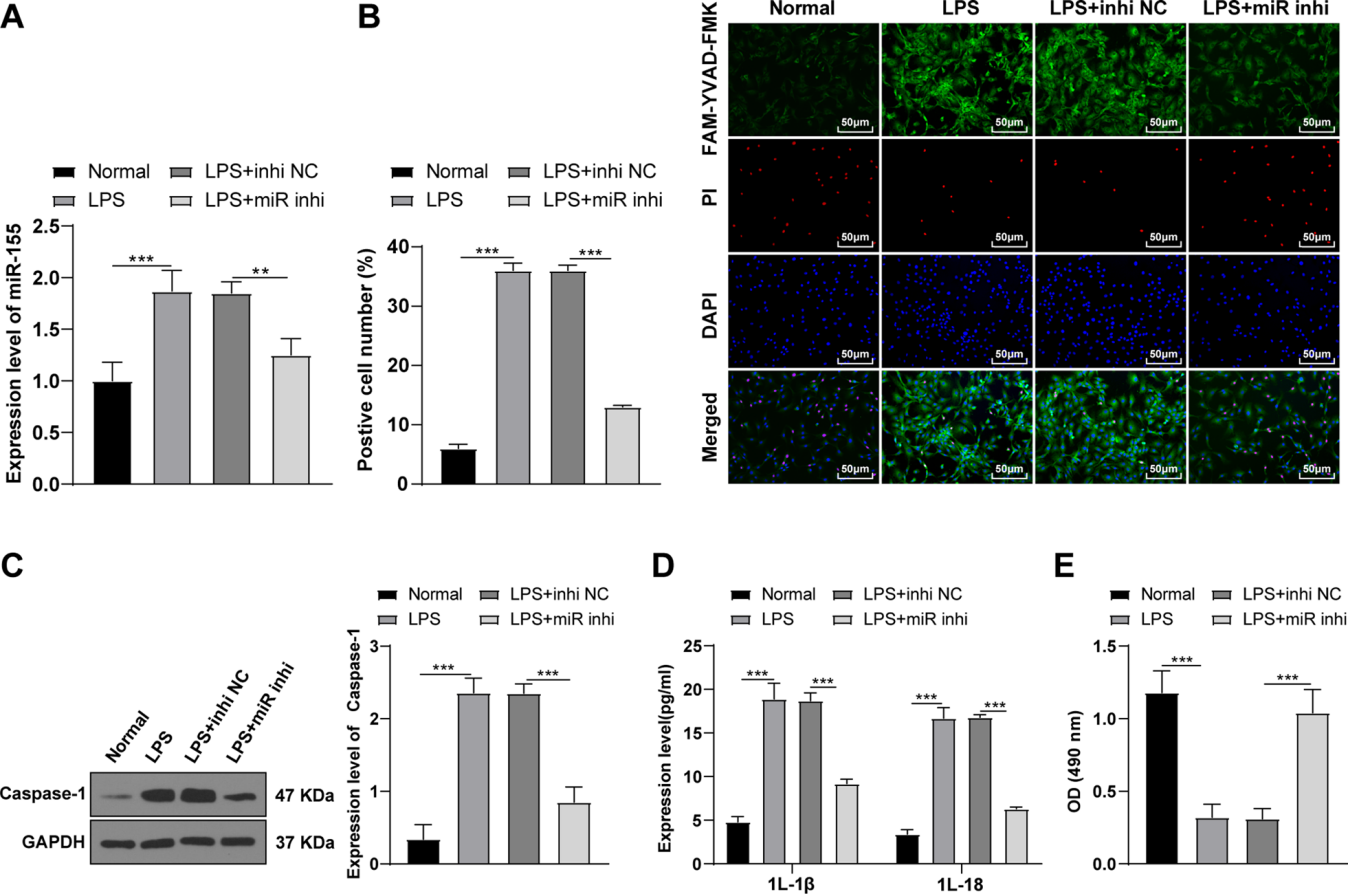
Knockdown of miR-155 inhibited LPS-induced KOA chondrocyte pyroptosis. The miR-155 inhibitor or NC was transfected into the chondrocytes on the basis of the LPS-induced KOA chondrocyte inflammatory model. **A** The expression of miR-155 was detected using RT-qPCR; **B** The cell pyroptosis was detected using the FAM-FLICA Caspase-1 Detection Kit; **C** The level of Caspase-1 was detected by WB; **D** The levels of the inflammatory factors IL-1β and IL-18 were detected by ELISA; **E** The cell viability was detected using the MTT method. The cell experiment was repeated 3 times. The data were expressed as mean ± standard deviation. One-way ANOVA was used for data comparisons among multi-groups. Independent t test was adopted for data comparisons between two groups and one-way ANOVA was employed for data comparisons among multi-groups. Tukey’s multiple comparisons test was used for the post hoc test. **P < 0.01, ***P < 0.001, ***P < 0.001

Pyroptosis is closely related to inflammatory diseases and plays an important role in arthritis [26]. Inhibition of chondrocyte pyroptosis can improve arthritis [26]. To explore whether miR-155 could regulate LPS-induced KOA chondrocyte pyroptosis, miR-155 was silenced in LPS-induced KOA chondrocytes. The miR-155 expression in chondrocytes was detected using RT-qPCR. Compared with the LPS + inhi NC group, miR-155 was downregulated in the LPS + miR inhi group (*P* < 0.05) (Fig. 2A), indicating that miR-155 was successfully knocked down. Subsequently, the pyroptosis level was detected using the FAM-FLICA Caspase-1 detection kit. Compared with the normal group, the pyroptotic chondrocytes were increased in the LPS group, while knockdown of miR-155 significantly diminished pyroptotic chondrocytes (all *P* < 0.05) (Fig. 2B). In addition, the pyroptosis-related protein Caspase-1 was detected by WB. Compared with the normal group, the level of Caspase-1 was increased in the LPS group, while knockdown of miR-155 significantly decreased Caspase-1 (all *P* < 0.05) (Fig. 2C). Furthermore, the levels of inflammatory factors in cells were detected using the ELISA kits. Compared with the normal group, the levels of IL-1β and IL-18 were increased in the LPS group, while knockdown of miR-155 significantly reduced the release of pro-inflammatory factors (all *P* < 0.05) (Fig. 2D). In addition, the cell viability was detected using the MTT kit. The viability of mouse KOA chondrocytes was significantly decreased after LPS induction, while knockdown of miR-155 promoted cell viability (*P* < 0.05) (Fig. 2E). Briefly, knockdown of miR-155 could inhibit LPS-induced KOA chondrocyte pyroptosis.

### miR-155 targeted SMAD2

**Fig. 3:**
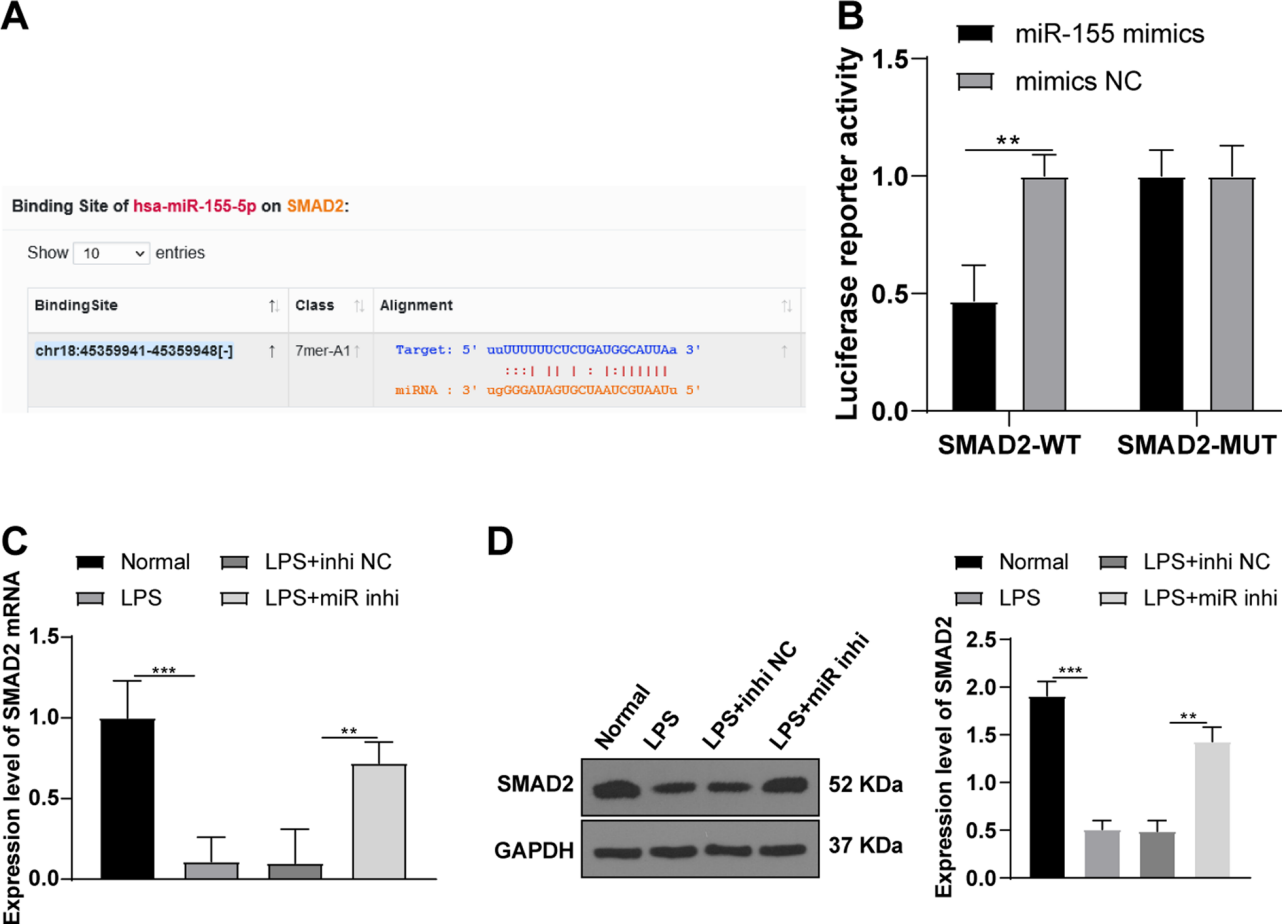
miR-155 targeted SMAD2. **A** The binding sites between miR-155 and SMAD2 WT 3ʹUTR were predicted using the Starbase; **B** luciferase activity detected by dual-luciferase assay; **C** The mRNA level of SMAD2 was detected using RT-qPCR; **D** The protein level of SMAD2 was detected by WB. The cell experiment was repeated 3 times. The data were expressed as mean ± standard deviation. One-way ANOVA was used for data comparisons among multi-groups. Independent t test was adopted for data comparisons between two groups and one-way ANOVA was employed for data comparisons among multi-groups. Tukey’s multiple comparisons test was used for the post hoc test. **P < 0.01, ***P < 0.001

The binding sites between miR-155 and SMAD2 were predicted using the online database (http://starbase.sysu.edu.cn/agoClipRNA.php?source=mRNA) (Fig. 3A). Furthermore, dual-luciferase assay showed that compared with the normal mouse knee chondrocytes co-transfected with mimics NC and SMAD-WT, the luciferase activity was decreased in the chondrocytes co-transfected with miR-155 mimics and SMAD-WT (*P* < 0.01), while the luciferase activity wasn’t significantly changed in the chondrocytes transfected with SMAD-MUT (Fig. 3B). A previous study shows that SMAD2 is vital in arthritis and the proliferation and migration of chondrocytes can be inhibited upon SMAD2 knockdown [19]. In addition, the mRNA and protein levels of SMAD2 in chondrocytes were detected by RT-qPCR and WB (Fig. 3C–D). Compared with the normal group, SMAD2 levels were decreased in the LPS group, while knockdown of miR-155 significantly increased SMAD2 (all *P* < 0.05). In short, miR-155 targeted SMAD2.

### Knockdown of SMAD2 partially averted the effects of miR-155 silencing on inhibiting KOA chondrocyte pyroptosis

**Fig. 4:**
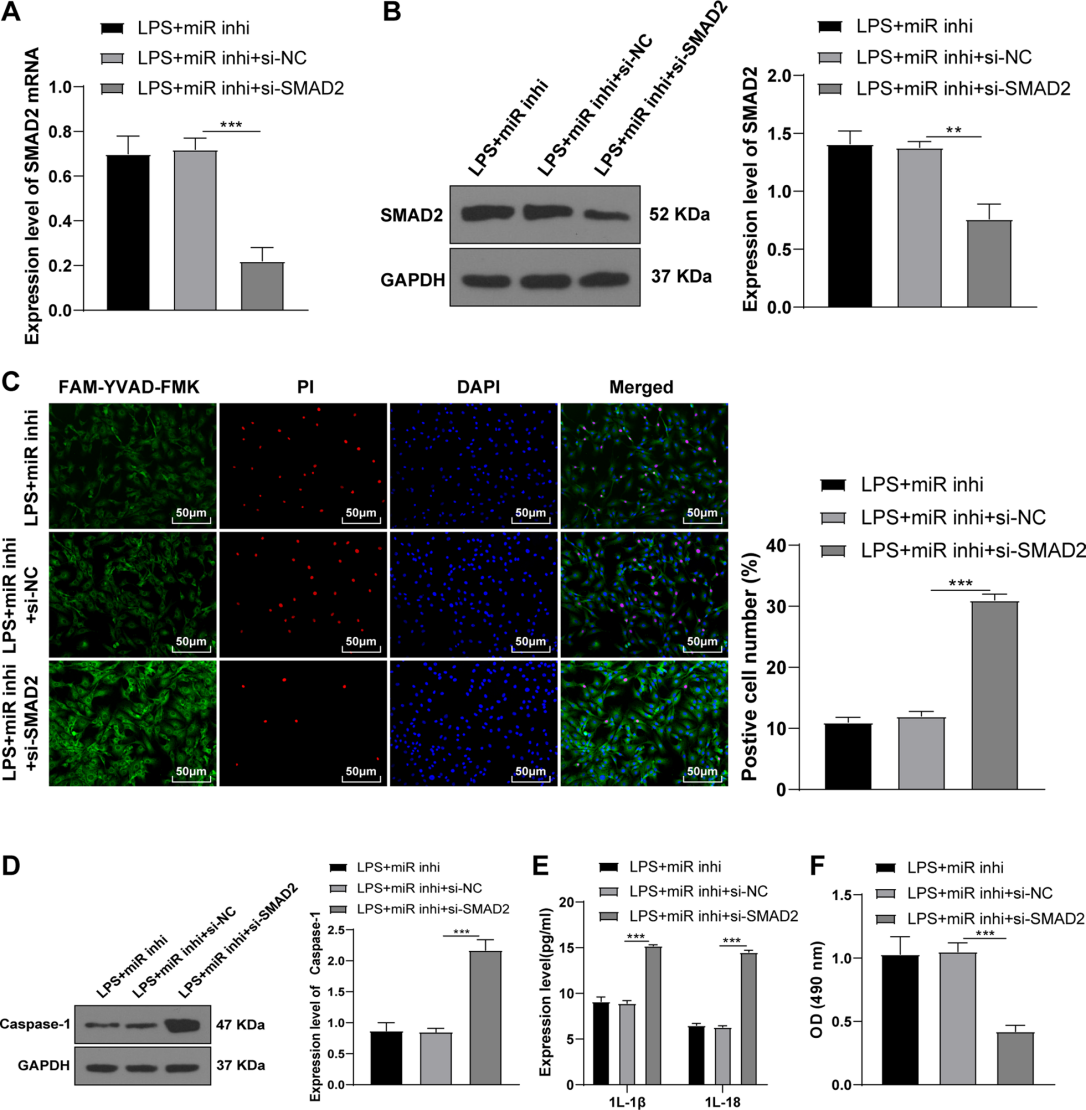
Knockdown of SMAD2 partially reversed the effects of miR-155 silencing on inhibiting KOA chondrocyte pyroptosis. **A** The mRNA level of SMAD2 was detected using RT-qPCR; **B** The protein level of SMAD2 was detected by WB; **C** The cell pyroptosis was detected using the FAM-FLICA Caspase-1 detection kit; **D** The level of Caspase-1 was detected by WB; **E** The levels of inflammatory factors IL-1β and IL-18 were detected by ELISA; **F** The cell viability was detected using the MTT method. The cell experiment was repeated 3 times. The data were expressed as mean ± standard deviation. One-way ANOVA was used for data comparisons among multi-groups. Tukey’s multiple comparisons test was used for the post hoc test. **P < 0.01, ***P < 0.001

To further verify whether miR-155 regulated KOA chondrocyte pyroptosis by targeting SMAD2, miR-155 was knocked down and SMAD2 was silenced in LPS-induced KOA chondrocytes through cell transfection. Compared with the LPS + miR inhi + si-NC groups, SMAD2 was significantly decreased in the LPS + miR inhi + si-SMAD2 group (all *P* < 0.05) (Fig. 4A-B). Compared with the LPS + miR inhi + si-NC group, the number of pyroptotic chondrocytes was increased (Fig. 4C) and Caspase-1 was increased (Fig. 4D) (all *P* < 0.05) in the LPS + miR inhi + si-SMAD2 group. In addition, the levels of inflammatory factors were significantly increased (Fig. 4E) and cell viability was decreased (Fig. 4F) (all *P* < 0.05) after knockdown of miR-155 and silencing of SMAD2. Altogether, silencing SMAD2 partially annulled the effects of miR-155 knockdown on inhibiting KOA chondrocyte pyroptosis.

### Knockdown of miR-155 inhibited the activation of the NLRP3 pathway through SMAD2

**Fig. 5:**
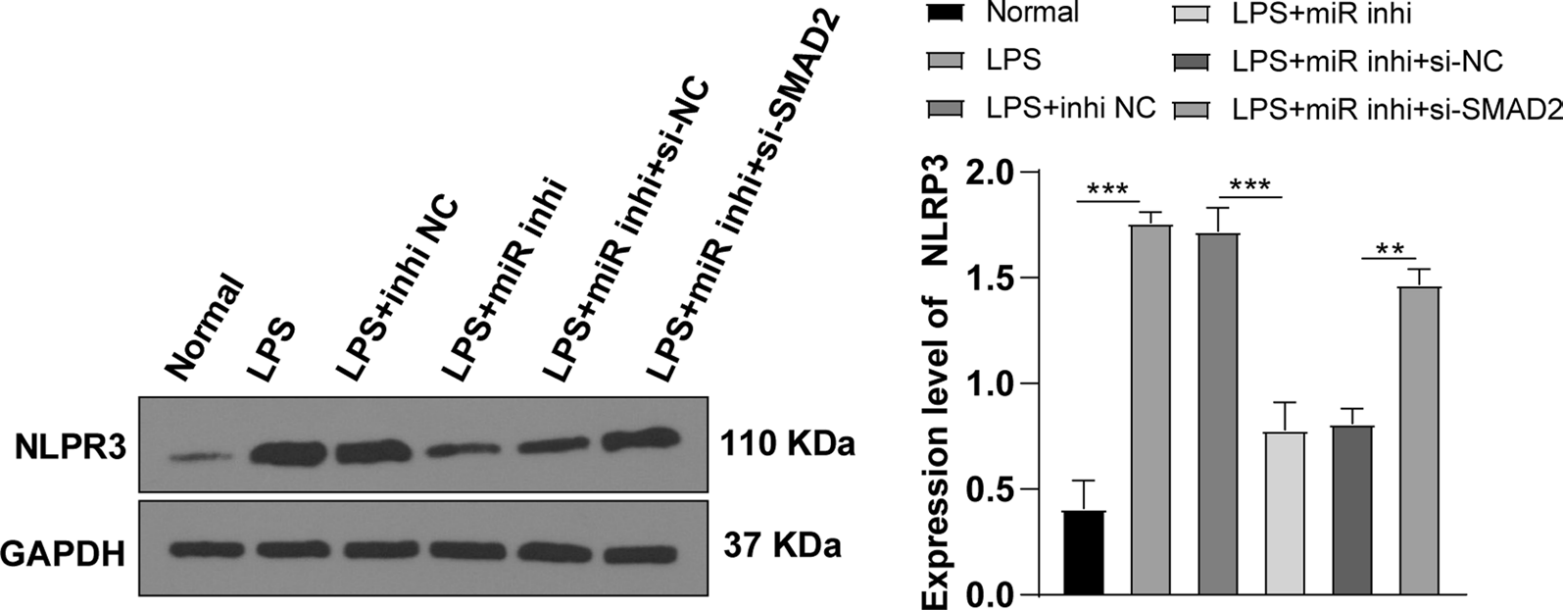
miR-155 inhibited the activation of the NLRP3 pathway through SMAD2. The level of NLRP3 in the pathway was detected by WB. The cell experiment was repeated 3 times. The data were expressed as mean ± standard deviation. One-way ANOVA was used for data comparisons among multi-groups. Tukey’s multiple comparisons test was used for the post hoc test. **P < 0.01, ***P < 0.001

The activation of the SMAD2/3 pathway can inhibit the transcription of NLRP3, thus reducing cell pyroptosis [22]. To further explore whether miR-155 inhibited the activation of the NLRP3/Caspase-1 pathway through SMAD2, the level of NLRP3 protein was detected by WB. Compared with the normal group, NLRP3 was significantly increased after LPS induction (*P* < 0.05) (Fig. 5). Compared with the LPS + miR inhi and LPS + miR inhi + si-NC groups, NLRP3 was increased in the LPS + miR inhi + si-SMAD2 group. Overall, miR-155 inhibited the activation of the NLRP3/Caspase-1 pathway through SMAD2.

### Knockdown of miR-155 inhibited KOA chondrocyte pyroptosis via the SMAD2/NLRP3/Caspase-1 axis

**Fig. 6:**
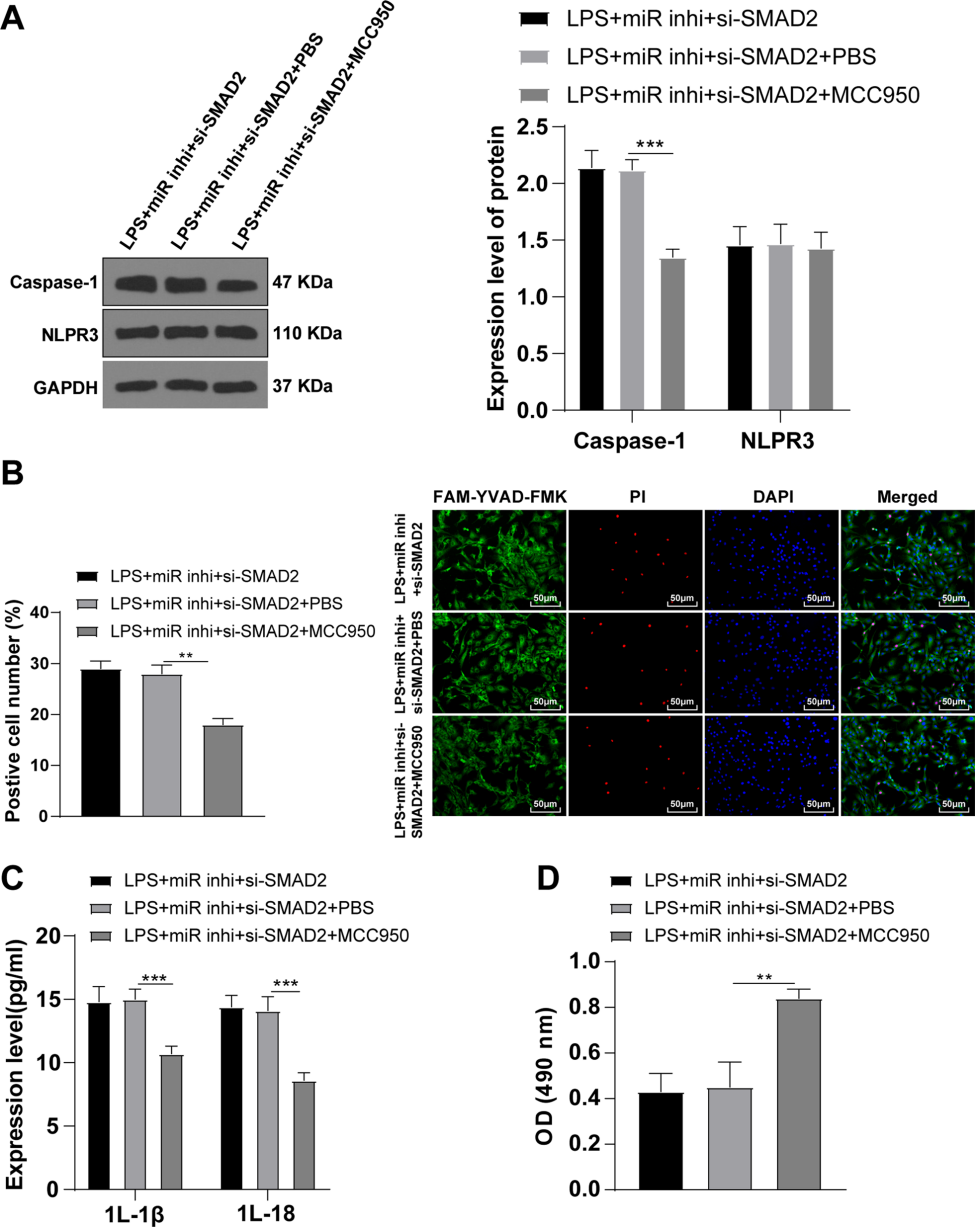
Knockdown of miR-155 inhibited KOA chondrocyte pyroptosis via the SMAD2/NLRP3/Caspase-1 axis. **A** The levels of NLRP3 and Caspase-1 were detected by WB; **B** The cell pyroptosis was detected using the FAM-FLICA Caspase-1 Detection Kit; **C** The levels of the inflammatory factors IL-1β and IL-18 were detected by ELISA; **D** The cell viability was detected using the MTT method. The cell experiment was repeated 3 times. The data were expressed as mean ± standard deviation. One-way ANOVA was used for data comparisons among multi-groups. Tukey’s multiple comparisons test was used for the post hoc test. **P < 0.01, ***P < 0.001

To further verify whether miR-155 inhibited KOA chondrocyte pyroptosis via the SMAD2/NLRP3/Caspase-1 axis, the cells were treated with NLRP3 inhibitor MCC950 based on the LPS + miR inhi + si-SMAD2 group. The levels of pyroptosis-related protein and inflammatory factors were detected by WB and RT-qPCR. The level of NLRP3 wasn’t changed apparently and Caspase-1 was significantly decreased (Fig. 6A) (*P* < 0.05). Compared with the LPS + miR inhi + si-SMAD2 group, the number of pyroptotic chondrocytes was decreased (Fig. 6B) (*P* < 0.05), the levels of IL-1β and IL-18 were decreased (Fig. 6C) (all *P* < 0.05), and cell viability was enhanced (Fig. 6D) (*P* < 0.05) after adding the NLRP3 inhibitor. Conjointly, knockdown of miR-155 could inhibit KOA chondrocyte pyroptosis via the SMAD2/NLRP3/Caspase-1 axis.

## Discussion

KOA is a peripheral joint disease with complex pathogenesis and multiple risk factors [1] and causes long-term disability and pain in the elderly population all over the world [27]. As the medium connecting bone and muscle, tendon transfers the force generated by muscle to bone to make the joint stabilize or move. Persistent knee varus or valgus may cause tendon injury, resulting in KOA [28]. Many growth factors and genes involved in tendon healing and tendon lesions can be regulated by miRNAs, and then play roles in the KOA occurrence and development [13]. A previous study has demonstrated the participation of miR-155 in KOA improvement [29]. This study illustrated that miR-155 knockdown inhibited KOA chondrocyte pyroptosis by targeting SMAD2 and inhibiting the NLRP3/Caspase-1 pathway.

miR-155-5p can serve as the potential therapeutic target for KOA [6]. Our results demonstrated significantly upregulated miR-155 expression in LPS-induced chondrocytes. It is consistent that LPS induction causes the increase of miR-155 expression in chondrocytes [17]. Pyroptosis is triggered by the inflammasomes after the activation of a variety of the inflammatory stimulations, such as improper LPS, which plays a role in knee OA [30]. To study whether miR-155 could regulate LPS-induced pyroptosis in KOA chondrocytes, we knocked down miR-155 in KOA chondrocytes after LPS induction. Our results elicited that pyroptotic chondrocytes were increased after LPS induction, while decreased after miR-155 knockdown. Pyroptosis has long been identified as the monocyte death mediated by Caspase-1 in response to the certain bacterial insults [31]. Our results demonstrated that Caspase-1 level in KOA chondrocytes was increased after LPS induction while decreased after miR-155 knockdown. Caspase-1 activation causes IL-1β and IL-18 maturation and the induction of pyroptosis [11]. In accordance, IL-1β and IL-18 levels were increased in KOA chondrocytes after LPS induction, while decreased after miR-155 knockdown; cell viability was decreased after LPS induction while increased after miR-155 knockdown. Similarly, miR-155 silencing decreases *Porphyromonas gingivalis*-induced macrophage pyroptosis rate [18]. But the regulation of miR-155 in chondrocyte pyroptosis has not studied so far. In summary, our results initially highlighted that miR-155 knockdown inhibited KOA chondrocyte pyroptosis induced by LPS.

To further study the downstream mechanism of miR-155, we predicted the target genes of miR-155 using the online database. Then, SMAD2 was identified. The binding relationship was verified by dual-luciferase assay. Additionally, SMAD2 level was decreased in KOA chondrocytes, while increased after miR-155 knockdown. It is consistent with that SMAD2 expression is downregulated in OA [20]. In brief, miR-155 targeted SMAD2. To verify whether miR-155 regulate KOA chondrocyte pyroptosis by targeting SMAD2, we knocked down SMAD2 in LPS-induced KOA chondrocyte with silenced miR-155 expression. Our results elicited that pyroptotic chondrocytes, Caspase-1 and IL-1β and IL-18 levels were increased and cell viability was decreased in KOA chondrocytes after both silencing miR-155and SMAD2 expression. Consistently, knockdown of SMAD2 inhibits proliferation and migration of chondrocytes in OA [19]. In conclusion, knockdown of SMAD2 partially reversed the effects of miR-155 silencing on inhibiting KOA chondrocyte pyroptosis.

The SMAD2/3 pathway activation inhibits NLRP3 transcription and reduces cell pyroptosis in hypoxic cardiomyocytes [22]. To study whether miR-155 inhibited the NLRP3/Caspase-1 pathway through SMAD2, we detected the NLRP3 level. Our results demonstrated that NLRP3 pathway was activated in LPS-induced KOA chondrocytes, inhibited after miR-155 knockdown, and activated again after further SMAD2 knockdown. In conclusion, miR-155 knockdown inhibited the NLRP3/Caspase-1 pathway through SMAD2. To verify whether miR-155 inhibited KOA cardiomyocyte pyroptosis via the SMAD2/NLRP3/Caspase-1 axis, we added NLRP3 inhibitor MCC950 in the cardiomyocytes with silenced miR-155 and SMAD2. Expectedly, NLRP3 level wasn’t changed, Caspase-1 level, pyroptotic chondrocytes, and IL-18 levels were decreased and cell viability was increased after NLRP3 inhibitor treatment. Consistently, knockdown of NLRP3 inhibits pyroptosis in synoviocytes from OA joints [23]. In brief, miR-155 knockdown inhibited KOA chondrocyte pyroptosis via the SMAD2/NLRP3/Caspase-1 axis.

In summary, this study revealed that miR-155 inhibited KOA chondrocyte pyroptosis by targeting SMAD2 and inhibiting the NLRP3/Caspase-1 pathway. These results are helpful to provide new ideas and new directions for the treatment for KOA from the genetic perspective. However, this study only verified that miR-155 inhibited LPS-induced KOA chondrocyte pyroptosis via the SMAD2/NLRP3/Caspase-1 axis in vitro, which was lack of animal experiments and clinical verification. In addition, the upstream lncRNAs regulating miR-155, other KOA-related miRNAs or other related target genes and pathways regulated by miR-155 need to be studied in the future.

## Data Availability

All the data generated or analyzed during this study are included in this published article.

## Abbreviations

KOA: Knee osteoarthritis
LPS: Lipopolysaccharide
OA: Osteoarthritis
IL-1β: Interleukin 1 beta
miRNAs: MicroRNAs
SMAD2: SMAD family member 2
TGF-β: Transforming growth factor β
NLRP3: Nucleotide-binding domain-like receptor protein3
DMEM: Dulbecco’s modified eagle medium
FBS: Fetal bovine serum
PBS: Phosphate buffer saline
BSA: Bovine serum albumin
DAB: 2,4-Diaminobutyric acid
RT-qPCR: Reverse transcription quantitative polymerase chain reaction
WB: Western blot
BCA: Bicinchoninic acid
SDS-PAGE: Sodium dodecyl sulfate–polyacrylamide gel electrophoresis
PVDF: Polyvinylidene fluoride
TBST: Tris buffered saline-Tween 20
ELISA: Enzyme-linked immunosorbent assay
MTT: 3-(4,5-Dimethylthiazol-2-yl)-2,5-diphenyltetrazolium bromide
DMSO: Dimethyl sulfoxide
OD: Optical density
PI: Propidium iodide
WT: Wild type
MUT: Mutant
ANOVA: Analysis of variance
